# Lack of association between *Toxoplasma gondii* infection and hypertensive disorders in pregnancy: a case–control study in a Northern Mexican population

**DOI:** 10.1186/1756-3305-7-167

**Published:** 2014-04-03

**Authors:** Cosme Alvarado-Esquivel, Fernando Vázquez-Alaníz, Ada A Sandoval-Carrillo, José M Salas-Pacheco, Jesús Hernández-Tinoco, Luis Francisco Sánchez-Anguiano, Oliver Liesenfeld

**Affiliations:** 1 Faculty of Medicine and Nutrition, Juárez University of Durango State, Avenida Universidad S/N, Durango, Dgo 34000, Mexico; 2 Hospital General de Durango, Secretaría de Salud, Avenida 5 de febrero y Norman Fuentes, Durango, Dgo 34000, Mexico; 3 Institute for Scientific Research “Dr. Roberto Rivera Damm”, Juárez University of Durango State, Avenida Universidad S/N, Durango, Durango 34000, Mexico; 4 Institute for Microbiology and Hygiene, Campus Benjamin Franklin, Charité Medical School, Hindenburgdamm 27, Berlin D-12203, Germany; 5 Present address: Roche Molecular Diagnostics, Pleasanton, CA, USA; 6 Laboratorio de Investigación Biomédica, Facultad de Medicina y Nutrición, Avenida Universidad S/N, Durango 34000, Dgo, Mexico

**Keywords:** *Toxoplasma gondii*, Seroprevalence, Preeclampsia, HELLP syndrome, Eclampsia, Infection, Epidemiology

## Abstract

**Background:**

The outcome of pregnancy is often threatened by hypertension disorders, i.e. eclampsia. Rate of infection with the protozoan parasite *Toxoplasma gondii* can be as high as 80% in pregnant women, and infection acquired during pregnancy can lead to fetal death. Very little is known about a potential association between infections, i.e. those with *Toxoplasma gondii,* and hypertensive disorders during pregnancy.

**Methods:**

Through a case–control study design, we investigated the presence of anti-*Toxoplasma* IgG and anti-*Toxoplasma* IgM antibodies in 146 pregnant women suffering from hypertensive disorders (cases) and 146 age-matched normotensive pregnant women (controls) attending a public hospital in Durango City, Mexico. Obstetric and blood pressure characteristics from cases and controls were also obtained.

**Results:**

Seroprevalence of anti-*Toxoplasma* IgG antibodies and IgG titers did not differ significantly in controls (8/146; 5.5%) and cases (9/146; 6.2%). Anti-*Toxoplasma* IgM antibodies were found in 2 (1.2%) controls and none of the cases. Seroprevalence of *T. gondii* in controls (5.5%) was similar to seroprevalences found in patients with mild preeclampsia (4/27: 14.8%), severe preeclampsia (5/95: 5.3%), eclampsia (0/16: 0%) and HELLP syndrome (hemolysis, elevated liver enzymes, and low platelet count) (0/8: 0%) (*P* = 0.23).

**Conclusions:**

Our results suggest that latent infection with *T. gondii* is not associated with hypertensive disorders in pregnant women in Northern Mexico. Further studies with larger sample sizes are needed to elucidate the association of infection with *T. gondii* with hypertensive disorders in pregnancy.

## Background

Hypertensive disorders in pregnancy cause maternal and perinatal morbidity and mortality [1,2]. About 8.5 million cases of preeclampsia are reported worldwide yearly [3]. The overall estimates of incidence in 40 countries including the 6 World Health Organization regions (African, European, Americas, Eastern Mediterranean, South-East Asia, and Western Pacific) were 4.6% and 1.4% of all deliveries for preeclampsia and eclampsia, respectively [4]. In Mexico, eclampsia and preeclampsia are recognized as direct causes of maternal deaths [5]. The etiology and primary pathology of preeclampsia remain elusive [1]. The role of infection in preeclampsia remains controversial. A body of evidence supports a link between preeclampsia and maternal infection [6,7]. For instance, in a systematic review of epidemiological studies, researchers found that any bacterial or viral infection was associated with a two-fold higher risk of developing preeclampsia compared to women without infection [6]. Furthermore, in a meta-analysis of 49 studies, urinary tract infection and periodontal disease during pregnancy were associated with an increased risk of preeclampsia [7]. More recent studies have recognized infections with high risk human papilloma virus [8], *Chlamydia trachomatis*[9], periodontitis [10], as well as *Chlamydia pneumoniae*[11] and cytomegalovirus IgG seropositivity [12] as risk factors for preeclampsia. On the other hand, some studies have not found an association of preeclampsia with infection with cytomegalovirus [13,14], *Chlamydia pneumonia*, *Herpes simplex virus* 2 [14], or respiratory tract infection [15]. HBsAg carriage [16] has even been found to be associated with a reduced incidence of preeclampsia.

Primary infection with *T. gondii* during pregnancy represents a risk for congenital disease [17,18]. The association of infection with *T. gondii* and preeclampsia has been insufficiently studied. Therefore, we performed a case–control study in Northern Mexico to determine the association between *T. gondii* infection and preeclampsia among pregnant women attending the Department of Gynecology and Obstetrics in a secondary-care public hospital in Durango City, Mexico.

## Methods

### Study design and study populations

Through a case–control study design, we studied the association of *T. gondii* infection with hypertensive disorders in pregnant women in Durango City, Mexico from November 2011 to September 2013. We used a 1:1 ratio for matching. Cases and controls were matched by age, gender, attending hospital, and residence.

#### Women with hypertensive disorders in pregnancy

Inclusion criteria for cases were: 1) women with 22–42 weeks of pregnancy suffering from hypertensive disorders and proteinuria attended in the Department of Gynecology and Obstetrics of a public secondary-care hospital (General Hospital) in Durango City, Mexico; and 2) who agreed to participate in the study. Hypertensive disorders included: 1) mild preeclampsia (blood pressure ≥140/90 mmHg on 2 occasions, at least 6 hours apart, and proteinuria of ≥300 mg/24 hours); 2) severe preeclampsia (blood pressure ≥160/110 mmHg on 2 occasions, at least 6 hours apart, and proteinuria of ≥5 g/24 hours); 3) eclampsia (hypertension, proteinuria and seizures); and 4) HELLP syndrome (hypertension, proteinuria and presence of hemolytic anemia, elevated liver enzymes and low platelet count). As a sampling strategy, all eligible women attended during the study period were invited to participate. In total, 146 cases accepted to participate in the study. All patients resided in Durango City.

#### Control subjects

Inclusion criteria for controls were: 1) pregnant women without hypertensive disorders, nephropathy, or diabetes before or early during pregnancy attended in the same Department of Gynecology and Obstetrics of The General Hospital in Durango City as cases; 2) normal pregnancy with normal blood pressure levels (systolic blood pressure <140 mmHg and diastolic blood pressure <90 mmHg); 3) without any underlying disease; and 4) who agreed to participate in the study. In total, 146 controls were included in the study. Controls were selected days after cases were included in the study.

Clinical data obtained from the patients (cases and controls) included age, number of pregnancies, deliveries, cesarean sections, miscarriages and stillbirths, week of pregnancy, history of preeclampsia, systolic and diastolic blood pressures, and mean arterial pressure.

### Ethical aspects

This study was approved by the Institutional Ethical Committee of The General Hospital of the Secretary of Health in Durango City, Mexico. The purpose and procedures of the study were explained to all participants, and a written informed consent was obtained from all of them.

### Laboratory tests

A blood sample of about 7 mL was obtained by venipuncture from the median cubital vein of each participant. Blood was centrifuged and serum samples were obtained and kept frozen at −20°C until analyzed. Sera were analyzed by qualitative and quantitative methods for anti-*T. gondii* IgG antibodies with the commercially available enzyme immunoassay kit “*Toxoplasma* IgG” (Diagnostic Automation Inc., Calabasas, CA, USA). Anti-*T. gondii* IgG antibody levels were expressed as International Units (IU)/ml, and a result equal to or greater than 8 IU/ml was considered positive. Sera positive for anti-*T. gondii* IgG antibodies were further analyzed for anti-*T. gondii* IgM antibodies by the commercially available enzyme immunoassay “*Toxoplasma* IgM” kit (Diagnostic Automation Inc., Calabasas, CA, USA). The cut off for anti-*T. gondii* IgM antibody positivity was obtained by multiplying the absorbance of the calibrator by the factor (0.40) recommended in the kit. We have used similar cut-offs for positivity to IgG and IgM antibodies in epidemiological studies in the region [19,20]. All tests were performed following the instructions of the manufacturer.

### Statistical analysis

Results were analyzed with the aid of Epi Info versions 3.5.4 and 7, and SPSS 15.0 (SPSS Inc. Chicago, Illinois). For calculation of the sample size, we used a 95% confidence level, a power of 80%, a 1:1 proportion of cases and controls, a reference seroprevalence of 6.1% [21] as the expected frequency of exposure in controls, and an odds ratio of 3.3. The result of the sample size calculation was 139 cases and 139 controls. A number of appropriate statistical methods for the study design [22] were used. Age values among the groups were compared by the paired student’s *t* test. For comparison of the frequencies of seropositivity to *T. gondii* and obstetric characteristics between cases and controls, the McNemar’s test was used. Odds ratio (OR) and 95% confidence interval (CI) were calculated by the Mantel-Haenszel analysis. We used the Pearson’s chi-squared test and the two-tailed Fisher’s exact test (when values were less than 5) to assess the association of *T. gondii* seropositivity and clinical and obstetric characteristics of the women studied. Statistical significance was set at a *P* value less than 0.05.

## Results

Of the 146 cases included in the study, 27 suffered from mild preeclampsia, 95 from severe preeclampsia, 16 from eclampsia, and 8 from HELLP syndrome. The mean age of the cases was 23.51 ± 6.41 years (range: 15–39 years) and the mean age of the controls was 23.44 ± 6.17 (range: 15–39). There was no statistically significant difference in age between cases and controls (*P* = 0.92).

Anti-*T. gondii* IgG antibodies were found in 8 (5.5%) of 146 controls and in 9 (6.2%) of 146 cases. There was not difference in *T. gondii* infection among case control pairs (Table 1). Of the 8 anti-*T. gondii* IgG positive control patients, 5 (3.4%) had IgG levels higher than 150 IU/ml, and 3 (2.1%) between 8 to 99 IU/ml. In comparison, of the 9 anti-*T. gondii* IgG positive cases, 6 (4.1%) had IgG levels higher than 150 IU/ml, and 3 (2.1%) between 8 to 99 IU/ml. Anti-*T. gondii* IgG levels in controls were similar to those in cases (OR = 1.20; 95% CI: 0.36-3.93; *P* = 0.76). Anti-*T. gondii* IgM antibodies were found in 2 (1.4%) controls but none were found in the cases in this study.

**Table 1:** **Distribution of *T. gondii *** **infection among case control pairs**

**Case**	**Control**		***Total***	***OR [95% CI]***	***p-value***
	**Exposed (*T. gondii *** **infection)**	**Unexposed (No *T. gondii *** **infection)**			
Exposed (*T. gondii* infection)	8	1	9		
Unexposed (No *T. gondii* infection)	0	137	137		
***Total***	8	138	146	3.0 [0.12-73.64]	1

With respect to clinical characteristics, seroprevalence of *T. gondii* in controls (5.5%) was similar to the seroprevalence found in cases with mild preeclampsia (4/27: 14.8%), severe preeclampsia (5/95: 5.3%), eclampsia (0/16: 0%) and HELLP syndrome (0/8: 0%) (*P* = 0.23). Of the obstetric characteristics, seropositivity to *T. gondii* was not associated with the deliveries, cesarean sections, miscarriages or a history of preeclampsia. In contrast, seroprevalence of *T. gondii* infection was significantly (*P* = 0.01) higher in women with more than one pregnancy (14/150: 9.3%) than in women with only one pregnancy (3/142: 2.1%). In addition, women with a history of stillbirths had a significantly (*P* = 0.009) higher (2/3: 66.7%) seroprevalence of *T. gondii* infection than women without such history (15/289: 5.2%). On the other hand, comparison of obstetric characteristics in cases and controls pairs showed that preeclampsia was positively associated with a history of preeclampsia and cesarean sections and negatively associated with having more than one pregnancy and a history of deliveries (Table 2).

**Table 2:** Distribution of obstetric characteristics among case control pairs

**Characteristic**	**Case**	**Control**		***Total***	***OR [95% CI]***	***p-value***
**Pregnancy history**		Exposed (More than 1 pregnancy)	Unexposed (One pregnancy)			
	Exposed (More than 1 pregnancy)	71	0	71		
	Unexposed (One pregnancy)	8	67	75		
	***Total***	79	67	146	0.05 [0.00-0.70]	0.01
						
**History of deliveries**		Exposed (History of delivery)	Unexposed (No history of delivery)			
	Exposed (History of delivery)	65	0	65		
	Unexposed (No history of delivery)	13	68	81		
	***Total***	78	68	146	0.03 [0.00-0.40]	0.001
						
**Cesarean sections**		Exposed (Cesarean section)	Unexposed (No Cesarean section)			
	Exposed (Cesarean section)	36	66	102		
	Unexposed (No Cesarean section)	0	44	44		
	***Total***	36	110	146	133.0 [8.23-2148.67]	<0.0001
						
**History of miscarriage**		Exposed (Miscarriage)	Unexposed (No miscarriage)			
	Exposed (Miscarriage)	21	4	25		
	Unexposed (No miscarriage)	0	121	121		
	***Total***	21	125	146	9.0 [0.48-167.17]	0.17
					
**History of stillbirth**		Exposed (Stillbirth)	Unexposed (No stillbirth)			
	Exposed (Stillbirth)	0	3	3		
	Unexposed (No stillbirth)	0	143	143		
	***Total***	0	146	146	7.0 [0.36-135.52]	0.31
					
**History of preeclampsia**		Exposed (Preeclampsia)	Unexposed (No preeclampsia)			
	Exposed (Preeclampsia)	6	15	21		
	Unexposed (No preeclampsia)	0	124	124		
	***Total***	6	139	145	31.0 [1.85-518.11]	0.0004

## Discussion

At present, very little is known about the role of infections in hypertension disease during pregnancy. This study was performed to investigate a potential association of infection with the protozoan parasite *T. gondii* with hypertensive disorders in pregnancy. Infections with *T. gondii* cause an increase in dopamine [23,24], and it is known that dopamine increases the blood pressure [25]. However, none of the serological markers of *T. gondii* infection, i.e., presence of anti-*T. gondii* IgG and IgM antibodies, and levels of anti-*T. gondii* IgG antibodies used in the present study correlated with hypertensive disorders in the pregnant women studied. In addition, seropositivity to *T. gondii* was also observed in similar frequencies between women with a history of preeclampsia and women without such history. Therefore, our results indicate that seropositivity to *T. gondii* is not likely to substantially contribute in the etiology of hypertensive disorders in pregnancy in our population.

Only two studies have investigated a potential association between infection with *T. gondii* and hypertension disorders, none of which observed an apparent association [11,26]. In a serological study in pregnant women in Norway, researchers found that women seronegative for *Herpes simplex virus* type 2, cytomegalovirus, and Epstein Barr virus had an increased risk for developing preeclampsia while seronegativity to *T. gondii* did not [26]. In a study in Turkey, seroprevalence of anti-*T. gondii* antibodies were similar in 54 preeclampsia women and 54 healthy pregnant women [11]. However, such studies only reported qualitative results on the seropositivity rate of infection in preeclampsia patients. In the present study, we performed a quantitative determination of anti-*T. gondii* IgG antibodies. Our results suggest that antibody concentrations were not related with hypertension in pregnant women. We studied mostly patients suffering from severe preeclampsia and small subgroups of hypertension disease including mild preeclampsia, eclampsia and HELLP syndrome. Therefore, the association of infection with *T. gondii* with mild preeclampsia, eclampsia and HELLP syndrome should be further investigated with larger sample sizes. There are still some questions to answer about the role of *T. gondii* infection in hypertension disease in pregnancy. In a case control study, treatment of pregnant women with spiramycin, a macrolide antibiotic administered to women with suspected infection before 18 weeks of pregnancy to reduce the rate of transmission of the parasite to the fetus [17] - reduced the incidence of pregnancy-induced hypertension [27]. In this study, researchers studied a cohort of 417 pregnant women treated with spiramycin because of seroconversion for *T. gondii* and 353 low-risk women who did not take any antibiotic during pregnancy, and observed that treated women had a lower risk of developing pregnancy-induced hypertension than untreated women. It is not clear whether such treatment directly impacted on the blood pressure; the authors hypothesized that the spiramycin treatment lowers the risk of developing preeclampsia probably by preventing the onset of infections that could complicate pregnancy [27]. Studies about the effects of dopamine on blood pressure in *T. gondii* infected subjects should be conducted.

## Conclusions

Results of the present study indicate that seropositivity to *T. gondii* was not associated with hypertensive disorders in pregnant women attending an obstetrics and gynecology department in Northern Mexico. However, due to the small sample size further studies with larger sample sizes are needed to elucidate the association of *T. gondii* infection with hypertension disease during pregnancy.

## Competing interests

The authors declare that they have no competing interests. Oliver Liesenfeld is Chief Medical Officer at Roche Molecular Systems.

## Authors’ contributions

CAE conceived and designed the study protocol, performed the laboratory tests and data analysis, and wrote the manuscript. FVA, AASC, and JMSP obtained the blood samples and clinical data, and performed the data analysis. JHT, LFSA and OL performed the data analysis and wrote the manuscript. All authors read and approved the final version of the manuscript.
